# V-ATPase-dependent repression of androgen receptor in prostate cancer cells

**DOI:** 10.18632/oncotarget.25641

**Published:** 2018-06-22

**Authors:** Yamhilette Licon-Munoz, Colleen A. Fordyce, Summer Raines Hayek, Karlett J. Parra

**Affiliations:** ^1^ Department of Biochemistry and Molecular Biology, School of Medicine, University of New Mexico Health Sciences Center, Albuquerque, New Mexico, 87131, USA

**Keywords:** prostate cancer, androgen receptor, V-ATPase, concanamycin, HIF-1α

## Abstract

Prostate Cancer (PCa) is the most commonly diagnosed cancer and the third leading cause of death for men in the United States. Suppression of androgen receptor (AR) expression is a desirable mechanism to manage PCa. Our studies showed that AR expression was reduced in LAPC4 and LNCaP PCa cell lines treated with nanomolar concentrations of the V-ATPase inhibitor concanamycin A (CCA). This treatment decreased PSA mRNA levels, indicative of reduced AR activity. V-ATPase-dependent repression of AR expression was linked to defective endo-lysosomal pH regulation and reduced AR expression at the transcriptional level. CCA treatment increased the protein level and nuclear localization of the alpha subunit of the transcription factor HIF-1 (HIF-1α) in PCa cells via decreased hydroxylation and degradation of HIF-1α. The addition of iron (III) citrate restored HIF-1α hydroxylation and decreased total HIF-1α levels in PCa cells treated with CCA. Moreover, iron treatment partially rescued CCA-mediated AR repression. Dimethyloxalylglycine (DMOG), which prevents HIF-1α degradation independently of V-ATPase, also decreased AR levels, supporting our hypothesis that HIF-1α serves as a downstream mediator in the V-ATPase-AR axis. We propose a new V-ATPase-dependent mechanism to inhibit androgen receptor expression in prostate cancer cells involving defective endosomal trafficking of iron and the inhibition of HIF-1 α-subunit turnover.

## INTRODUCTION

The luminal pH of intracellular compartments is highly controlled [1–3]. A critical enzyme involved in the process of pH regulation is the vacuolar (H^+^) – ATPase (V-ATPase). V-ATPase is a proton pump located at intracellular compartments of the endomembrane system (e.g., endosomes, lysosomes, Golgi-derived vesicles, clathrin-coated vesicles, secretory vesicles) and the plasma membrane of eukaryotic cells specialized for active proton secretion [4, 5]). V-ATPase is a multi-subunit complex that has 14 different subunits arranged in two functional domains. V_1_ is the catalytic domain on the cytosolic side of the membrane. It is composed of eight subunits (A_3_B_3_CDE_3_FG_3_H). The V_1_ domain hydrolyzes ATP and has three catalytic sites located at the interface of alternating subunits A and B. The V_o_ domain is the proton translocation domain and consists of six subunits (a, c, c´´, d, e and Ac45 in mammals). The V_o_ domain subunits c and c” form a proteolipid ring structure that rotates when protons are transferred across the membrane [1, 2, 5]. V-ATPases are frequently overexpressed in tumors and tumor cell lines [4, 6, 7], suggesting that proper control of organelle pH is essential for cellular health. V-ATPase has been reported to aid in tumor invasion and migration [4, 8–12], drug resistance to chemotherapy [13–16], and cell death [17–20].

Prostate Cancer (PCa) is the most commonly diagnosed cancer and the third leading cause of death for men in the United States [21]. Normal prostate cells and early stage PCa cells depend upon androgen activity for growth and survival [22–24]. Thus, androgen ablation is a common therapy for PCa, often resulting in the regression of androgen-dependent tumors. However, tumors frequently become androgen-independent and typically recur with increased metastatic ability [22–26]. These Ablation Resistant Prostate Cancer (ARPC) tumors lower the relative survival of patients. Suppression of androgen receptor (AR) expression is a desirable mechanism to manage PCa.

Hypoxia inducible factor 1 (HIF-1) is a transcription factor that regulates oxygen homeostasis [27], angiogenesis [28, 29], glucose metabolism [30], invasion [29, 31] and cell survival [29]. Hence, its regulation is important for cancer progression [32]. HIF-1 is overexpressed in PCa tumors and can be regulated by androgen activity [33]. HIF-1 is a heterodimer with two subunits: HIF-1β, which is constitutively expressed, and HIF-1α, which is hydroxylated and targeted for degradation by the von Hippel-Lindau (VHL) ubiquitin ligase in normoxic conditions [32, 34, 35]. In carcinogenesis, HIF-1 has a dual function: it can induce the expression of genes that promote both hypoxic adaptation (e.g., VEGF, GLUT-1, PGK, LDH-A [27–30]) and apoptosis (e.g., NIX, NIP3, p53 [34, 36]).

In this study, we propose a new mechanism to inhibit AR expression in prostate cancer cells. We show that AR expression is reduced in PCa cell lines treated with the V-ATPase inhibitor concanamycin A (CCA). We demonstrate that this effect is a result of decreased HIF-1α hydroxylation and turnover that is linked to V-ATPase-dependent defects in the endosomal traffic of iron. This pathway can be used to modulate AR expression and eventually lead to the development of novel therapeutic tools for prostate cancer.

## RESULTS

### V-ATPase is required for androgen receptor expression

**Figure 1:**
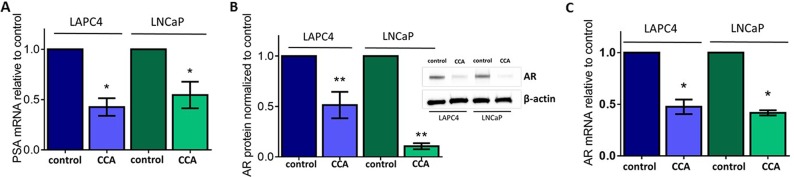
V-ATPase inhibition represses androgen receptor in prostate cancer cell lines LAPC4 and LNCaP cell lines, which express Androgen Receptor (AR), were exposed to vehicle control (0.01% DMSO) or 10 nM of the V-ATPase inhibitor Concanamycin A (CCA) for 24 hours. **(A)** Prostate Specific Antigen (PSA) mRNA levels were evaluated using quantitative real time PCR (qPCR). Bars represent the mean PSA mRNA level relative to matched control (n = 3) in LAPC4 (blue) and LNCaP (green) cells. **(B)** Western blots and densitometry analysis of LAPC4 and LNCaP whole cell lysates were used to monitor AR protein levels using β-actin as a loading control; insert shows representative western blot. Bars represent the mean AR protein level relative to matched control (n = 5). **(C)** AR mRNA levels were evaluated using qPCR. Bars represent the mean AR mRNA level relative to matched control (n = 3). A-C error bars represent standard error of the mean, ^*^ indicates p < 0.05, ^**^ indicates p < 0.01 compared to control as determined by Student's t-test.

The prostate-specific antigen (PSA) is a serine protease that cleaves semenogelins in the seminal coagulum. PSA is the most commonly used PCa biomarker. In an earlier study, we reported that treatment with the V-ATPase inhibitor bafilomycin A (BAF, 10 nM) decreased mRNA levels of PSA in the PCa cell line LNCaP [12]. We validated those studies by showing that a second V-ATPase inhibitor, concanamycin A (CCA), also inhibited PSA mRNA expression in the LAPC4 and LNCaP PCa cell lines. We observed a significant reduction in PSA mRNA levels (50 - 60%) after a 24 hour treatment with 10 nM CCA (Figure 1A). We verified that cell survival was not compromised by this dose of CCA (Supplementary Figure 1).

PSA gene expression is regulated by the transcriptional activity of the Androgen Receptor (AR) [37]. Thus, we next asked whether V-ATPase inhibition impaired AR function. We first examined AR protein expression in whole cell lysates of LAPC4 and LNCaP cells treated with CCA for 24 hours. Immunoblot analyses using a monoclonal antibody against AR showed significantly decreased AR protein levels (~90% less in LNCaP and ~49% less in LAPC4) in cells exposed to the V-ATPase inhibitor compared to untreated controls (Figure 1B).

We measured AR mRNA to determine whether the reduction in AR protein was associated with reduction in AR mRNA expression. Using qPCR, we showed that cells exposed to CCA expressed significantly lower levels of AR mRNA than the untreated cells (~50% reduction) (Figure 1C). An important difference between the LAPC4 and LNCaP cell lines is that LAPC4 cells express wild-type AR, while LNCaP cells express a mutant allele of the AR (T877A). Whereas AR mRNA expression was comparable in both cell lines treated with CCA (Figure 1C), our results showed significantly lower AR protein expression levels in LNCaP cells (Figure 1B), suggesting that the mutant AR was less stable after CCA exposure. Together, these results indicate that V-ATPase function is required for AR expression and link V-ATPase function to the PSA-AR axis in prostate cancer.

### Androgen receptor expression is dependent upon endo-lysosomal pH homeostasis

**Figure 2:**
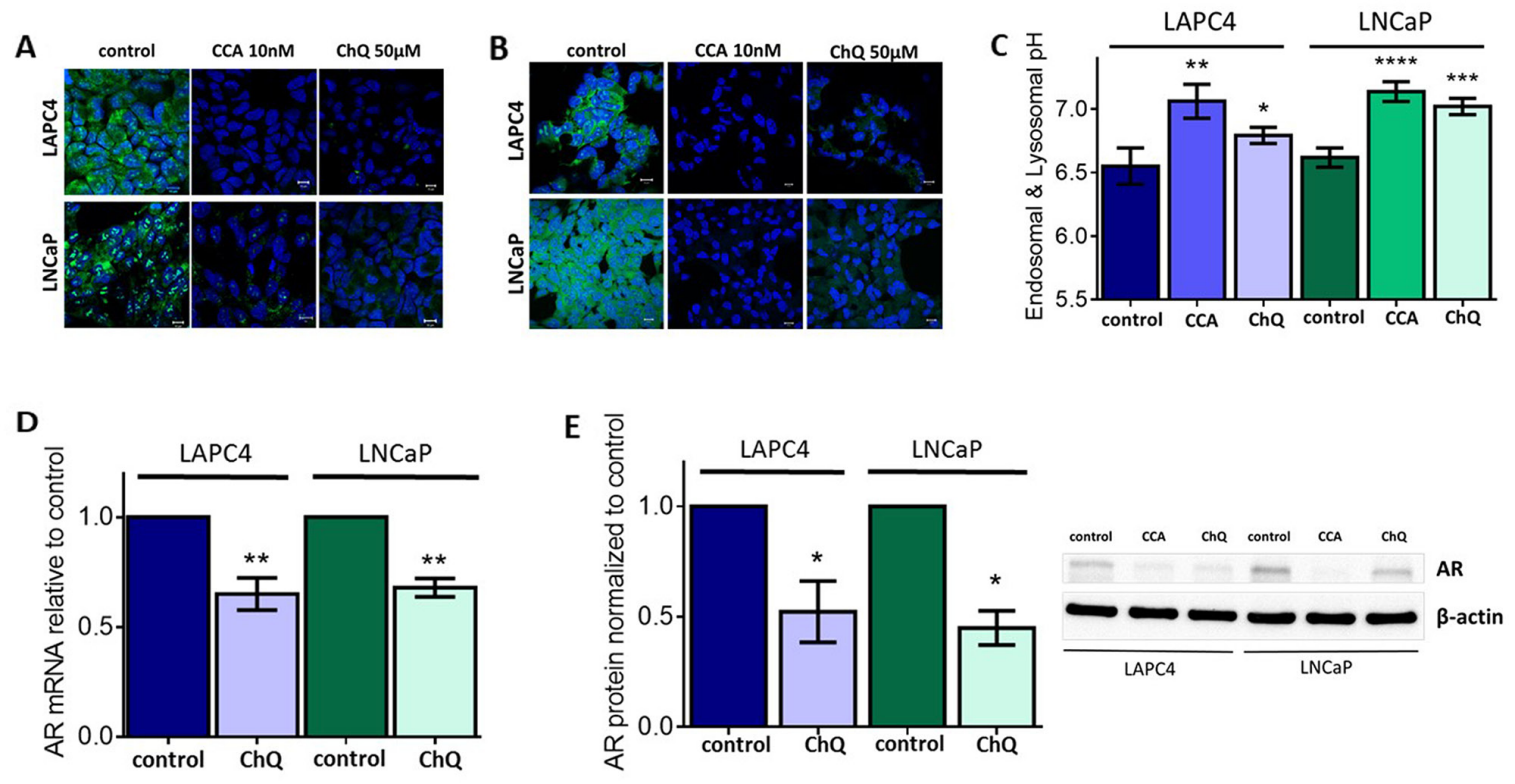
Endo-lysosomal alkalinization is sufficient to reduce androgen receptor expression levels **(A-B)** LAPC4 (top panel) and LNCaP (bottom panel) cell lines were plated on glass coverslips, allowed to attach, and then treated with 0.01% DMSO (control), 10nM concanamycin A (CCA), or 50 μM chloroquine (ChQ) for 1hr (A) or 24h (B). Coverslips were stained with 1 μM acridine orange (green) for 30 minutes and analyzed using fluorescent confocal microscopy. DAPI (blue) was used as nuclear marker. Scale bar =10 μM. **(C)** LAPC4 and LNCaP cells were incubated with HPTS and then treated with 0.01% DMSO (control), 10 nM CCA, or 50 μM ChQ for 1 hour. Mean endosome and lysosome pH is shown ± SE from 5-7 experiments. **(D)** AR mRNA levels (n=3) and **(E)** AR protein levels (n=3) were evaluated following treatment with vehicle control (0.01% DMSO) or 50 μM ChQ for 24 hours as described in Figure 1B-1C. (C-E) error bars represent standard error of the mean, ^*^ indicates p < 0.05, ^**^ indicates p < 0.01, ^***^ indicates p < 0.001, ^****^ indicates p < 0.0001 compared to control as determined by Mann Whitney test (C) or Student's t-test (D-E).

Inhibition of V-ATPase activity disrupts cellular pH homeostasis [2], because V-ATPase acidifies the lumen of organelles in the endomembrane system and affects cytoplasmic and extracellular pH [1, 2]. We used acridine orange staining to monitor V-ATPase-dependent pH alterations in acidic organelles. Acridine orange is a weak base that accumulates in acidic vesicles and emits fluorescence [38]. As expected, both acute treatment (1 hour, Figure 2A) and chronic treatment (24 hours, Figure 2B) with CCA decreased acridine orange accumulation in these organelles, suggesting that organelle acidification is defective upon V-ATPase inhibition in LAPC4 and LNCaP cells. To quantify the pH in these organelles, we used the pH-sensitive fluorescent dye, 8-Hydroxypyrene-1,3,6-trisulfonic acid (HPTS). HPTS enters the cell by endocytosis and accumulates in endosomes and lysosomes [39, 40]. The endo-lysosomal pH of both LAPC4 and LNCaP cells exposed to 1 hour of CCA treatment was significantly increased as compared to the untreated cells (6.55 ± 0.14 to 7.06 ± 0.13 in LAPC4 cells; 6.61 ± 0.07 to 7.13 ± 0.07 in LNCaP cells) (Figure 2C).

Chloroquine (ChQ) is a lysosomotropic amine that accumulates in acidic vesicles and increases the endo-lysosomal pH [41]. We treated our PCa cell lines with 50 μM ChQ to determine if aberrant pH homeostasis inhibits AR expression independently of V-ATPase. This dose of ChQ does not compromise cell survival (Supplementary Figure 1). Acridine orange staining was comparable in cells treated with 50 μM of ChQ and those cells treated with 10 nM CCA (Figure 2A–2B). Compared to control, the endo-lysosomal pH significantly increased (to 6.79 ± 0.06 in LAPC4 cells and to 7.02 ± 0.06 in LNCaP cells) after treatment with ChQ (Figure 2C). Notably, addition of 50 μM ChQ decreased AR mRNA (Figure 2D) and AR protein levels (Figure 2E). These results indicate that the reduction in AR expression following V-ATPase inhibition likely results from aberrant pH homeostasis rather than a direct effect of V-ATPase itself. These results also specifically link endo-lysosomal organelle pH to AR expression in prostate cancer cells. To our knowledge, this is the first report that links AR expression with endo-lysosomal pH homeostasis.

### V-ATPase inhibition impairs androgen receptor gene transcription

**Figure 3:**
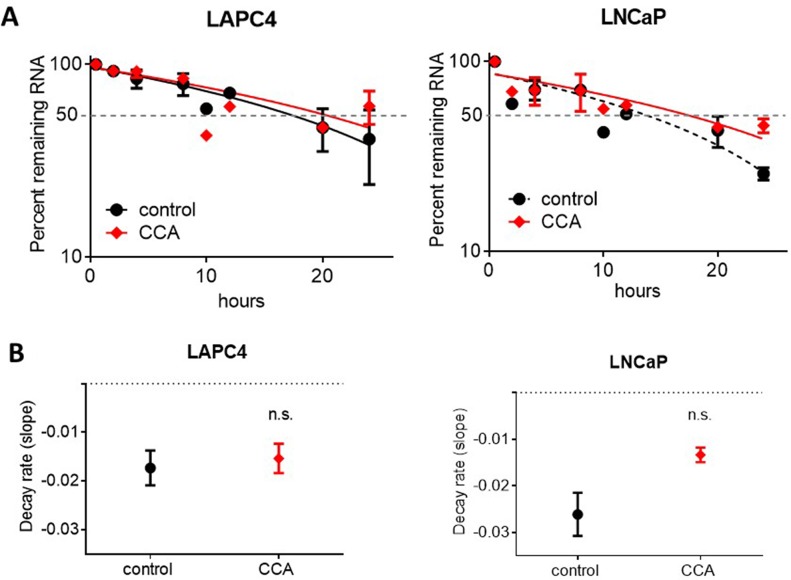
Androgen receptor mRNA degradation is not stimulated by V-ATPase inhibition LAPC4 and LNCaP cell lines were exposed to 5μg/ml actinomycin D and 0.01% DMSO (control, black circles) or 10 nM CCA (red diamonds). **(A)** Samples were collected at 0.5, 2, 4, 8, 10, 12, 20 and 24 hours and AR mRNA levels were monitored via qRT-PCR. Data are expressed as percent remaining mRNA at each time point relative to time 0. **(B)** Decay rates were calculated as the slope of the lines shown in Figure 3. A-B error bars represent standard error of the mean (n=3), n.s. indicates not significant (p > 0.05) compared to control as determined by Mann-Whitney test.

In an attempt to explain our noted decrease in AR expression, we determined whether treatment with CCA impaires AR mRNA stability and turnover. To accomplish this, we measured AR mRNA decay rates in LAPC4 and LNCaP cells treated with 10 nM CCA in the presence of the transcription inhibitor actinomycin D (5 μg/ml) [42, 43]. CCA treatment did not enhance the AR mRNA decay rate (LAPC4: control = −0.01730 ± 0.003565 hours vs. CCA = −0.01536 ±0.003021 hours; LNCaP: control = −0.02611 ± 0.004625 hours vs. CCA = −0.01333 ± 0.001572 fours) (Figure 3). Mann-Whitney analysis of AR mRNA decay rates showed that modest differences between control and CCA treatment were not statistically significant (LAPC4: *p* = 0.8; LNCaP: *p* = 0.1) (Figure 3B). We concluded that AR mRNA degradation was not stimulated by CCA treatment, and V-ATPase inhibition likely impairs transcription of the AR gene.

### HIF1α protein levels and translocation to the nucleus increase when V-ATPase is inactive

Transcription of the AR is tightly controlled. One pathway regulating AR gene expression involves the α subunit of the Hypoxia Inducible Factor-1 (HIF-1α) transcription factor [44–47]. Notably, in breast cancer cells lines, HIF-1α has been reported to repress transcription of the estrogen hormone receptor, ERα [48], and V-ATPase inhibition was reported to increase HIF-1α protein levels in several other cancer cell lines [49, 50]. We therefore asked whether V-ATPase inhibitors affect HIF-1α expression and stability in prostate cancer cells and whether HIF-1α may link V-ATPase and AR.

**Figure 4:**
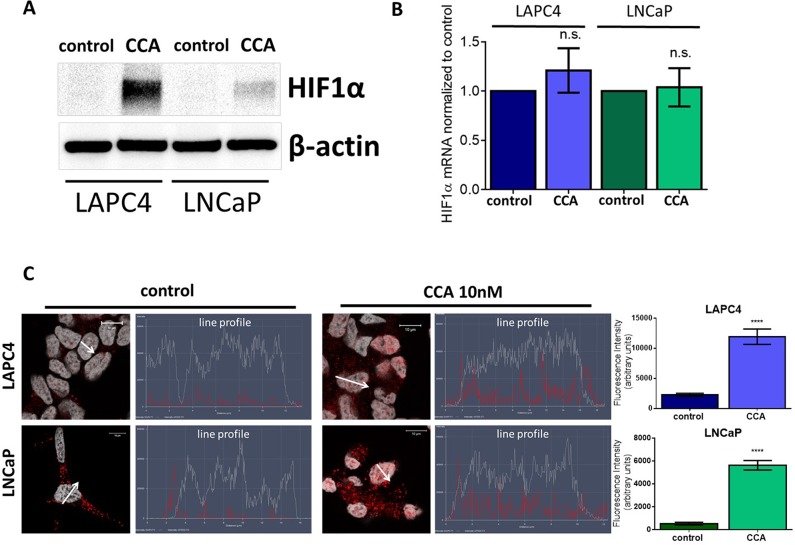
V-ATPase inhibition increases HIF1α protein levels and nuclear localization in prostate cancer cell lines LAPC4 and LNCaP cell lines were exposed to vehicle control (0.01% DMSO) or 10 nM CCA for 24 hours. **(A)** Western blots of whole cell lysates were used to monitor HIF-1α protein levels using β-actin as a loading control; image shows representative western blot (n ≥3). **(B)** HIF1α mRNA levels were evaluated using qPCR. Bars represent the mean HIF1α mRNA level relative to matched control (n = 4). **(C)** LAPC4 (top panel) and LNCaP (bottom panel) cell lines were plated on glass coverslips, allowed to attach, and then treated with 0.01% DMSO (control) or 10nM concanamycin A (CCA) for 24h. Coverslips were immunostained with an antibody against HIF-1α, labeled with AlexaFluor secondary antibody (red), and analyzed using fluorescent confocal microscopy. DAPI (gray) was used as nuclear marker. Co-localization was analyzed using confocal microscopy determining a line profile of fluorescence intensity. Arrow shows line profile x-axis. Scale bar =10 μM. Graphs show the mean fluorescence intensity of HIF-1α in the nucleus (n=10). (B-C) error bars represent standard error of the mean, n.s. indicates not significant (p > 0.05), ^****^ indicates p < 0.0001 compared to control as determined by Student's t-test (B) and Mann Whitney test (C).

To determine if CCA treatment alters HIF-1α expression, we first monitored HIF-1α protein levels. We analyzed whole cell lysates from LAPC4 and LNCaP cells treated with 10 nM CCA for 24 hours. Western blots showed more HIF-1α in cells exposed to CCA than in untreated control cells (Figure 4A). Notably, HIF-1α mRNA levels did not significantly change upon treatment with CCA (Figure 4B). These results suggest that V-ATPase inhibition enhances HIF-1α protein translation and/or stability and not HIF-1α transcription.

When active, HIF-1α translocates to the nucleus to act as a transcription factor [32, 34, 51]. Line profile analysis of fluorescent intensity shows higher levels (5-fold increase in LAPC4 and 10-fold increase in LNCaP) of HIF-1α nuclear localization in CCA-treated cells as compared to cells exposed to vehicle control (Figure 4C). Our results suggest that V-ATPase inhibition induces HIF-1α translocation to the nucleus.

### Loss of V-ATPase activity disrupts iron homeostasis and reduces downstream HIF-1α hydroxylation and degradation, leading to decreased androgen receptor expression

The observation that HIF-1α levels increase in the presence of CCA could be explained if V-ATPase inhibition prevents HIF-1α degradation. Under normoxic conditions, HIF-1α is rapidly turned over by a process that involves hydroxylation by prolyl hydroxylase, which targets HIF-1α for degradation by the proteasome [32, 34]. Prolyl hydroxylases require iron as a co-factor [32, 34], and notably, V-ATPase inhibition decreases endocytosis of the transferrin receptor (TfR) [11], thus lowering intracellular iron concentrations [52, 53].

**Figure 5:**
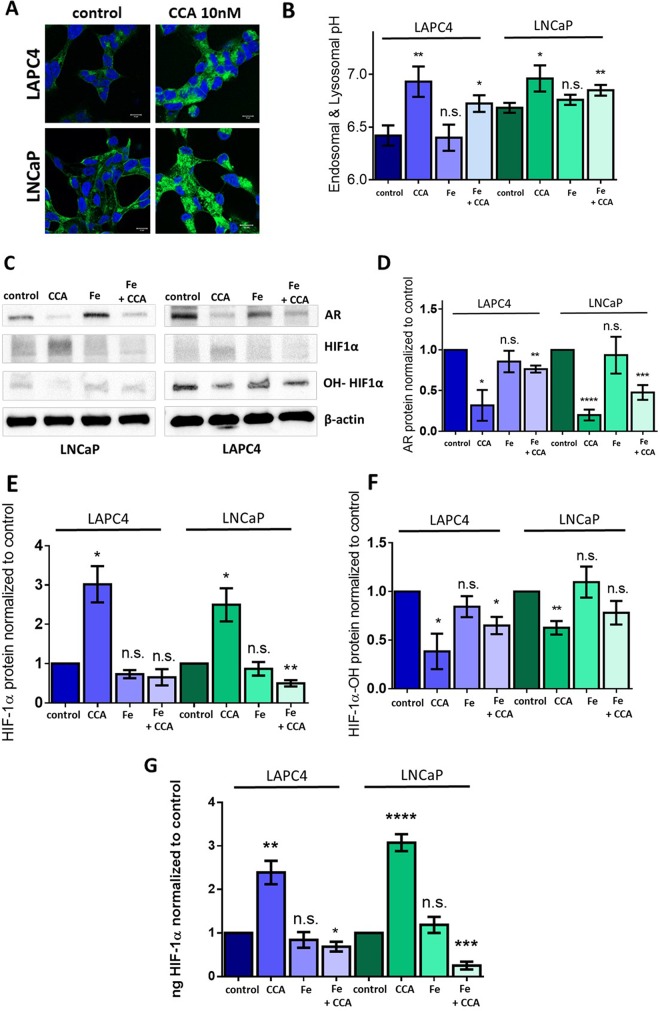
Iron (III) citrate treatment partially restores HIF-1α hydroxylation and androgen receptor expression in the face of V-ATPase inhibition **(A)** LAPC4 (top panel) and LNCaP (bottom panel) cell lines were plated on glass coverslips, allowed to attach, and then treated with 0.01% DMSO (control) or 10nM concanamycin A (CCA) for 24h. Coverslips were immunostained with an antibody against TfR, labeled with AlexaFluor secondary antibody (green), and analyzed using fluorescent confocal microscopy. DAPI (blue) was used as nuclear marker. Scale bar =10 μM. **(B)** LAPC4 and LNCaP cells were incubated with HPTS and then exposed to vehicle control (0.01% DMSO), 10 nM CCA, 500 μM Iron(III) citrate (Fe), or CCA and Fe together for 1 hour. Bars represent the mean endosome and lysosome pH (n=3-5). **(C)** LAPC4 and LNCaP cells were treated with conditions described in Figure 5B above for 24 hours. Western blots of whole cell lysates were used to monitor AR, HIF-1α, and hydroxylated HIF-1α (OH-HIF1α) protein levels using β-actin as a loading control; image shows representative western blot. **(D)** Densitometry analysis of AR western blots. Bars represent the mean AR protein level relative to matched control (n = 3-5). **(E)** Densitometry analysis of HIF-1α western blots. Bars represent the mean HIF-1α protein level relative to matched control (n = 3). **(F)** Densitometry analysis of hydroxylated HIF-1α (OH-HIF1α) western blots. Bars represent the mean HIF-1α protein level relative to matched control (n = 3-4). **(G)** ELISA quantification of HIF-1α in whole cell lysates. Bars represents mean ng HIF-1α normalized to control (n = 3-4). B; D-G error bars represent standard error of the mean, n.s. indicates not significant (p ≥ 0.05), ^*^ indicates p < 0.05, ^**^ indicates p < 0.01, ^***^ indicates p < 0.001, ^****^ indicates p < 0.0001 compared to control as determined by Mann-Whitney test (B) and Student's t-test (D-G).

In this study, exposure of PCa cell lines to CCA increased intracellular TfR signal (Figure 5A, green) and endo-lysosomal pH (Figure 5B) compared to control. This suggests that when V-ATPase is inactive, TfR accumulates in intracellular vesicles and endocytosis of iron is likely impaired. We hypothesized that these low intracellular iron concentrations would inactivate prolyl-4-hydroxylase, thus reducing HIF-1α hydroxylation and increasing HIF-1α stability and activity. To test our hypothesis, we attempted to use iron to rescue the HIF-1α-related phenotypes in PCa cells with decreased V-ATPase activity. LAPC4 and LNCaP cells were treated with 10 nM CCA and 500 μM iron (III) citrate separately or simultaneously for 24 hours, and whole cell lysates were analyzed via immunoblot (Figures 5C-5F) and ELISA (Figure 5G). Cells treated with iron alone mimicked untreated control cells. Cells treated with CCA alone showed increased levels of HIF-1α that correlated with decreased levels of hydroxylated HIF-1α compared to control (Figures 5C, 5E-5G). Iron rescued the effects of CCA on total HIF-1α protein and hydroxylated HIF-1α (Figures 5C, 5E-5G), indicating that normal HIF-1α turnover requires proper iron homeostasis, and V-ATPase inhibition disrupts this process. Iron also partially rescued AR gene expression in CCA-treated cells (2.3 times higher expression in LAPC4 cells and 2.4 times higher expression in LNCaP cells compared to CCA-treated cells without iron) (Figure 5D). These results indicate that HIF-1α acts as a transcriptional repressor of the AR gene in prostate cancer cell lines with decreased V-ATPase activity.

Notably, treatment with iron did not significantly rescue CCA-induced endo-lysosomal pH defects. Both cells treated with CCA alone and cells treated with CCA and iron (III) citrate together showed significantly elevated endo-lysosomal pH when compared to vehicle-treated control (Figure 5B). Thus, iron restores a step in the V-ATPase-to-AR pathway that is downstream of V-ATPase-mediated pH alterations.

**Figure 6:**
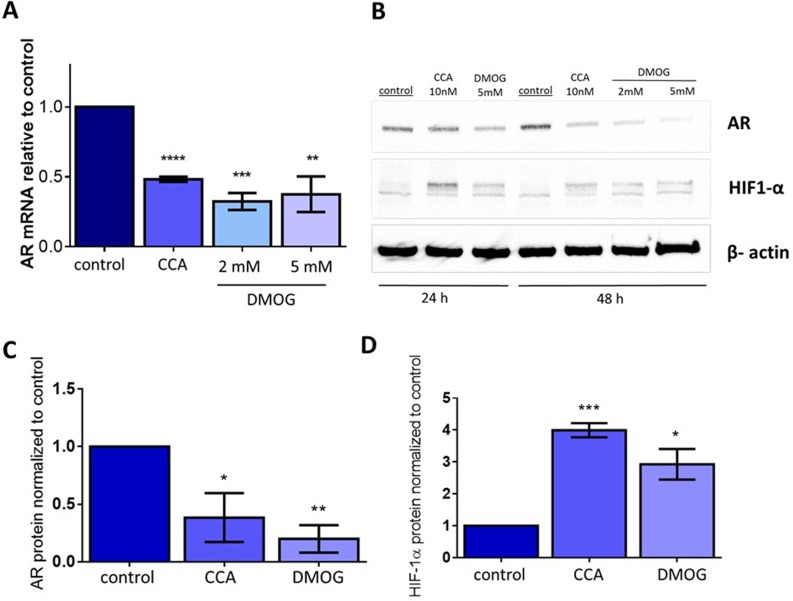
Stabilizing HIF1α independently of V-ATPase inhibition is sufficient to decrease androgen receptor expression LAPC4 cell lines were exposed to vehicle control (0.01% DMSO), 10 nM CCA, 2mM DMOG, or 5mM DMOG for 24-48 hours. **(A)** AR mRNA levels were evaluated using qPCR after 48 hours. Bars represent the mean AR mRNA level relative to matched control (n = 3). **(B)** Western blots of whole cell lysates were used to monitor AR and HIF-1α protein levels using β-actin as a loading control; image shows representative western blot. **(C)** Densitometry analysis of AR western blots. Bars represent the mean AR protein level relative to matched control (n = 3). **(D)** Densitometry analysis of HIF-1α western blots. Bars represent the mean HIF-1α protein level relative to matched control (n = 3). Error bars represent standard error of the mean, ^*^ indicates p < 0.05, ^**^ indicates p < 0.01, ^***^ indicates p < 0.001, ^****^ indicates p < 0.0001 compared to control as determined by Student's t-test.

We also asked whether preventing degradation of HIF-1α independently of V-ATPase would inhibit AR expression. Dimethyloxalylglycine (DMOG) is a cell permeable prolyl-4-hydroxylase inhibitor, which prevents HIF-1α hydroxylation and its subsequent degradation [54, 55]. We hypothesized that treatment with DMOG would mimic CCA-induced iron depletion and would thus increase HIF-1α protein levels and decrease AR expression. Indeed, after addition of DMOG, expression of AR mRNA (Figure 6A) and AR protein levels (Figure 6B and 6C) were reduced compared to control, while HIF-1α protein levels increased (Figure 6B and 6D). These changes were comparable to those induced upon treatment with CCA, confirming that the down-regulation of AR transcription seen during V-ATPase inhibition occurs via reduced hydroxylation and increased stabilization of HIF-1α.

## DISCUSSION

**Figure 7:**
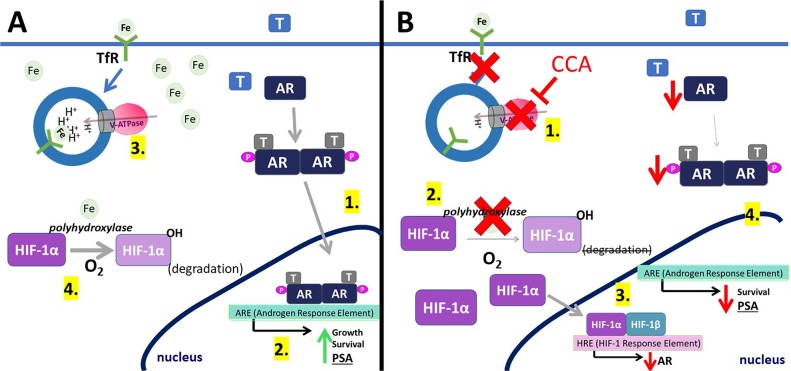
Model for V-ATPase inhibition-induced androgen receptor repression **(A)** (1) In control cells, testosterone (T) activates the androgen receptor (AR). This binding induces AR phosphorylation and dimerization. (2) AR dimers can bind to androgen-response elements (ARE) in the promoter regions of target genes, leading to cell growth, cell proliferation, and expression of PSA. (3) V-ATPase (pink and gray) generates and sustains the pH gradient required for proper receptor-mediated endocytosis. Transferrin Receptor (TfR) uses endocytosis to take iron (Fe) into the cell. (4) Iron is used as a co-factor in the hydroxylation of HIF-1α (HIF-1α-OH), which is then targeted for degradation. **(B)** (1) Concanamycin (CCA) treatment inhibits V-ATPase, leading to TfR-vesicle accumulation, which decreases intracellular iron. (2) Without iron, HIF-1α cannot be hydroxylated and is not degraded. HIF-1α can bind HIF-1β. (3) HIF-1α/β binds to HIF-1 response elements in the AR gene promoter, thereby decreasing AR expression. (4) Decreasing AR levels slows down steps (A1) and (A2), leading to less cell proliferation and less expression of PSA.

This study identified a novel downstream effector of V-ATPase in prostate cancer cells; our results show for the first time that androgen receptor expression is V-ATPase-dependent (Figure 1), and we present a cellular mechanism that links V-ATPase to AR expression and activity (Figure 7). To summarize, loss of V-ATPase activity causes alkalinization of endo-lysosomal compartments (Figure 2). This disrupted pH homeostasis negatively affects TfR endocytic trafficking (Figure 5) [2, 11], leading to reduced iron uptake [56]. Under low intracellular iron concentrations, HIF-1α hydroxylation is blocked (Figure 5), thus increasing its stability and function. HIF-1α is then free to translocate to the nucleus and down-regulate AR gene expression (Figures 4-6).

The most intrinsic function of V-ATPase is its ability to acidify intracellular compartments [1, 2]. Indeed, we demonstrated that during V-ATPase inhibition, it is these general pH alterations that mediate AR repression, rather than a V-ATPase-specific signaling pathway (Figure 2). Iron availability is a prime candidate effector pathway downstream of pH. In support of this hypothesis, TfR accumulates intracellularly after V-ATPase inhibition in PCa cells (Figure 5A), and aberrant TfR endocytic recycling results in iron depletion in several other cell lines [56, 57]. In the current study, we demonstrated that treatment with iron partially rescues AR expression in cells where V-ATPase is inhibited (Figure 5D). However, iron does not rescue the endo-lysosomal alkalinization (Figure 2 and 5B) or the defective TfR localization (data not shown) in CCA-treated cells. Thus, iron restores AR expression at a step downstream of V-ATPase–mediated luminal pH and membrane traffic defects. These findings also suggest that proper iron regulation can compensate in the face of disrupted pH elsewhere in the cell, clarifying the critical role that iron availability plays in androgen receptor function.

Specifically, iron appears to play a role in the HIF-1α degradative process (Figures 5C, 5E-5G). HIF-1 is a heterodimer composed of one regulatory α subunit and one β subunit [33]. HIF-1β is constitutively expressed, whereas the expression level of HIF-1α determines the extent of active HIF-1 (α/β) available to drive expression of pro-survival and pro-apoptotic genes [32, 51]. HIF-1α expression is maintained in part by regulated protein turnover. Prolyl hydroxylases hydroxylate HIF-1α, which can then bind the VHL ubiquitin ligase [34, 51], thereby targeting HIF-1α for degradation in the proteasome [51].

The HIF-1α hydroxylation reaction requires oxygen as a co-factor [34]. Therefore, under normoxic conditions, prolyl hydroxylases are active and HIF-1α is degraded rapidly (Figure 7A) [32]. In contrast, during hypoxia, HIF-1α cannot be hydroxylated and its degradation is blocked. Notably, iron also serves as co-factor for prolyl hydroxylase [34], and CCA-treated PCa cells under normoxic conditions display decreased HIF-1α hydroxylation and turnover (Figure 5C, and 5F) and increased HIF-1α translocation to the nucleus (Figure 4C); these effects are reversible following iron repletion (Figure 5C and 5F). Thus, V-ATPase inhibition mimics chronic hypoxia, because the resulting lack of intracellular iron constitutively increases HIF-1α, despite adequate cellular oxygen levels (Figure 7B).

Treatment of PCa cells with the prolyl hydroxylase inhibitor DMOG mimics the effect of V-ATPase inhibition, increasing HIF-1α levels (Figure 6B and 6D) and decreasing AR expression at both the mRNA (Figure 6A) and protein level (Figure 6B and 6C). These findings demonstrate the importance of HIF-1α as the molecular effector linking V-ATPase activity and androgen receptor expression in prostate cancer cells. In previous studies, V-ATPase inhibitors were shown to increase HIF-1α levels in a different PCa cell line, PC-3, as well as in other cancers [49, 50, 57]. Thus, this V-ATPase-HIF-1α pathway is not specific to prostate cancer. Rather, it is likely a general mechanism that can be exploited to manipulate HIF-1α levels. However, our studies are the first to link the V-ATPase-HIF-1α axis specifically to AR levels in prostate cancer.

Crosstalk of AR and HIF-1α in PCa has been previously reported [44–47]. However, to our knowledge, HIF-1α inhibition of AR gene expression has not been reported in the past, although HIF-1α has been shown to repress transcription of the carbamoyl phosphate synthetase-aspartate carbamoyltransferase-dihydroorotase gene in normal cells [58] and the estrogen receptor alpha gene in breast cancer cells [48]. The exact mechanism by which HIF-1α acts as a transcriptional repressor is unknown. It is possible that HIF-1α directly binds to the AR gene, thus inhibiting its expression. Alternatively, a molecule activated by HIF-1α (e.g. p53) may indirectly represses the AR [59, 60]. Further studies are required to dissect the exact nature of this mechanism.

Unexpectedly, co-incubation of CCA and iron significantly reduces HIF-1α protein levels to below even the untreated control (Figure 5E and 5G). An explanation for this phenomenon will require future study, but one intriguing possibility involves the interplay between androgen signaling and HIF-1α. Under normal conditions, when V-ATPase is functional and AR is present, baseline levels of HIF-1α protein expression are maintained in part by androgen signaling through the phosphatidylinositol 3′-kinase (PI3K) signaling pathway [61]. Perhaps this AR-androgen-PI3K-HIF-1α axis is not functional during CCA+Fe treatment, despite the recovery of AR expression (Figure 5C and 5D), because the signaling pathway requires functional V-ATPase and/or proper endo-lysosomal acidification. Since these processes fall upstream of iron rescue, they remain abnormal in CCA+Fe treated cells (Figure 5B), thus leading to reduced androgen-to-HIF-1α signaling and a drop in HIF-1α below the baseline levels preserved in control cells. This hypothesis is especially intriguing given the known interaction between PI3K and V-ATPase in mammalian cells, although thus far, the interaction appears unidirectional, with PI3K signaling modulating V-ATPase assembly and activity [62–64].

The V-ATPase-dependent decreases in AR expression outlined here are pertinent to both wild-type and mutant AR PCa cell lines and tumors. LAPC4 cells have a wild-type allele of the AR [65], whereas LNCaP cells have a T877A mutation in the ligand-binding domain of the AR [66]. This mutation has been reported in patients who have been treated with androgen ablation therapies [67], and the mutation prevents inhibition of AR activity, conferring resistance to anti-androgen treatments [22, 66]. Notably, in our studies, inhibition of V-ATPase effectively blocks expression of the AR-T887A allele in LNCaP cells (Figures 1, 2, 5). V-ATPase inhibitors may thus lead to new treatments relevant for patients that have PCa tumors containing mutant versions of AR, as V-ATPase inhibition represses AR activity at the transcriptional level, overriding any treatment resistance due to protein mutations.

Our studies with CCA support previous studies using a second V-ATPase inhibitor, bafilomycin (BAA). In those studies, treatment of PCa cells with BAA reduced PSA secretion [12], suggesting that AR transcriptional activity was inhibited. Both CCA and BAA are precomacolide antibiotics and share the same mechanism of action: these V-ATPase inhibitors bind to the V_o_ domain at the interface between the V_o_a and V_o_c subunits of V-ATPase [68], thereby inhibiting the ATP-driven rotation that is necessary for proton translocation across membranes. While this class of V-ATPase inhibitors shows promise in repressing AR expression in PCa cells, CCA and BAA target all V-ATPases indiscriminately of cell type, tissue, or organ [68], leading to detrimental side effects in the patient. Therefore, the development of a new generation of V-ATPase inhibitors directed specifically toward tumor tissue is critical for improving prostate cancer patient outcomes.

## MATERIALS AND METHODS

### Cell lines and conditions

LAPC4 cells (kind gift from Christopher M. Heaphy, PhD) were grown in ISCOVE's Modified Dulbecco's Medium containing 10% FBS and 1 nM R1881. LNCaP cells (purchased from ATCC) were cultured in RPMI-1640 media supplemented with 10% fetal bovine serum (FBS). Cells were authenticated using short tandem repeat profiling and were free of mycoplasma contamination. Cells were maintained at 37°C and 5% CO_2_ in a humidified atmosphere. Cells were exposed to vehicle (0.01% DMSO, SIGMA), concanamycin A (10 nM, Enzo Scientific), chloroquine (50 μM, Sigma), iron (III) citrate (500μM, Sigma), and/or dimethyloxalylglycine (2-5mM, Cayman Chemicals) for 24 hours unless otherwise indicated. Cell viability under certain treatments was assessed with Tetrazolium MTT (3-(4, 5-dimethylthiazolyl-2)-2, 5-diphenyltetrazolium bromide) Assays (ATCC) where indicated. All experiments were performed with cells less than 50 passage and with at least three biologically independent experiments.

### Quantitative real-time PCR (qPCR)

RNA was isolated using the Roche High Pure RNA isolation kit and reverse transcribed with the RETROscript^®^ cDNA kit. qRT-PCR was performed with SYBR Green I Mastermix on a Roche LightCycler 480 II. Analysis was performed using Δ/ΔCt method and expression of β-glucuronidase (GUSB) was used as an internal standard. Primer sequences are listed in Table 1.

**Table 1:** Primers used in qRT-PCR

Protein	Forward Primer 5′- 3′	Reverse Primer, 5′- 3′
GUSB	CTCATTTGGAATTTTGCCGATT	CCGAGTGAAGATCCCCTTTTTA
AR	ATGGGACCTGAGCTGTTGGAA	GTTCCAATGCAGGAAACTGCC
PSA	AGCCCCAAGCTTACCACCTG	TCAGGGGTTGGCCACGATGG

### Western blotting

Whole cell lysates (WCL) were prepared using RIPA buffer (25 mM Tris, 8 mM MgCl2, 1 mM DTT, 15% glycerol, 1% Triton X-100, and protease inhibitor cocktail (SIGMA)). Protein concentrations were determined using bicinchoninic acid assay (BCA) assay (Pierce) against a BSA standard curve. For each Western sample, 50- 100 μg total protein of WCL were diluted in 4X Laemmli Buffer and loaded on 8% polyacrylamide gels. Primary antibodies against AR (Santa Cruz Biotechnology), HIF-1α (Abcam and Proteintech (not shown)), hydroxyproline-HIF-1α (Millipore), and β-actin (SIGMA) were diluted in 5% milk in TBS-T at 1:1000 concentration. Immunoblots were imaged using the ChemiDocTM XRS workstation (BioRad). Densitometry quantification of western blot bands was performed using ImageJ software.

### pH measurements

#### Acridine orange

To qualitatively assess changes in pH of acidic vesicles, cells were incubated with acridine orange (SIGMA, 1 μM) for 30 minutes at 37^°C^, then incubated with 4′,6-Diamidino-2-phenylindole dihydrochloride (DAPI) (1 μg/ml; SIGMA) for 5 minutes. Cells were fixed on glass slides with 4% paraformaldehyde. Slides were imaged in Zeiss LSM 800 Airyscan Confocal microscope.

#### Endosome/Lysosome pH measurements

To quantitatively assess pH in endo-lysosomes, cells were incubated with 1 mM 8-Hydroxypyrene-1,3,6-trisulfonic acid (HPTS) (Life Technologies) for ~16 hours, then incubated with the drug of interest for 1 hour. Fluorescence was measured using a FluoroMax 4 spectrofluorometer (Horiba Jobin Yvon) with an excitation ratio of 458/405 nm at a fixed emission of 515 nm. The HPTS fluorescence excitation 458/405 ratio was converted to pH values by comparison to standard curves generated using known pH buffers (i.e., pH 5 to pH 7.4) analyzed by non-linear regression (i.e. exponential growth equation) (Supplementary Figure 2) [11, 39, 40].

### Immunocytochemistry

Cells were cultured on glass coverslips and were allowed to attach for 24 hours prior to the initiation of treatment. Cells were fixed with 4% paraformaldehyde following permeabilization with 0.02% TritonX-100. Cells were blocked with 5% goat serum (GS). Incubation with primary antibodies against HIF-1α (Abcam) and Transferrin Receptor (Invitrogen) was performed at 1:100 dilution in 5% GS for 1 hour. Cells were washed with phosphate buffer saline (PBS) and incubated for 30 minutes with the secondary fluorescent antibodies (AF488 and AF546, Invitrogen; 1:500 in 5% GS), then incubated with DAPI (1 μg/ml; SIGMA) for 5 minutes. Cells were washed with PBS and mounted onto microscope slides in mounting media. Slides were imaged in Zeiss LSM 800 Airyscan Confocal microscope. Co-localization was analyzed using confocal microscopy determining a line profile of fluorescence intensity. The average fluorescence intensity of several line profiles was obtained to analyze differences in HIF-1α nuclear localization in control versus CCA-treated cells. All steps were performed at room temperature.

### ELISA

Changes in HIF-1α of whole cell lysates were quantified using an ELISA HIF-1α kit (Thermofisher) according to the manufacturer's instructions.

### Statistical analysis

Non-paired Student's t-tests or Mann-Whitney tests were performed to determine statistical significance between control and experimental groups. Statistical analysis was performed using GraphPad Prism 5 software.

## SUPPLEMENTARY MATERIALS FIGURES


