# Supplementary material for: V-ATPase-dependent repression of androgen receptor in prostate cancer cells
Source: Oncotarget. 2018 Jun 22;9(48):28921–34. doi: 10.18632/oncotarget.25641 (PMC6034745; doi:10.18632/oncotarget.25641)
Supplement: Supplementary file 1 [file oncotarget-09-28921-s001.pdf]

V-ATPase-dependent repression of androgen receptor in prostate cancer cells

SUPPLEMENTARY MATERIALS

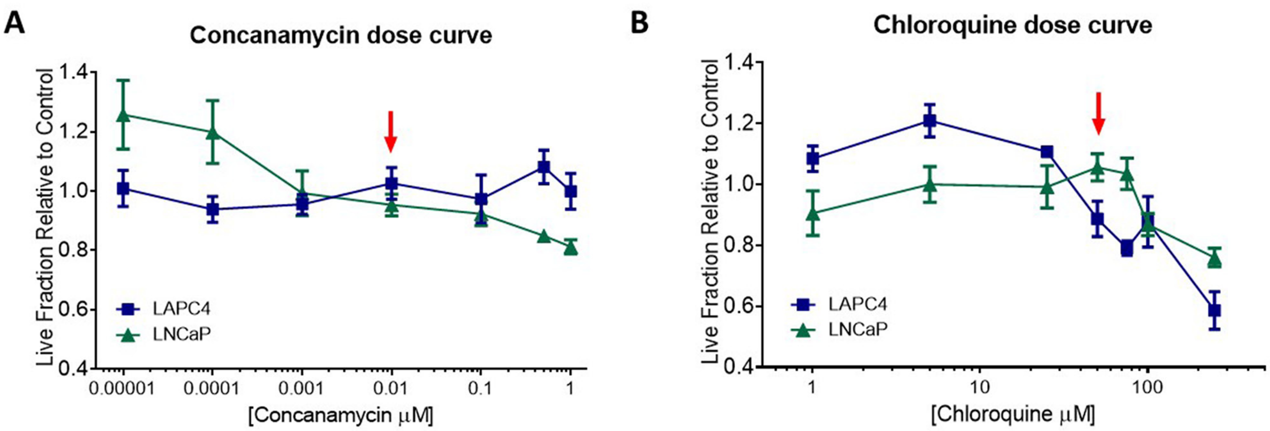

Supplementary Figure 1: Experimental doses of concanamycin A and chloroquine do not decrease survival of prostate cancer cell lines. LAPC4 cells (blue squares) and LNCaP cells (green triangles) were exposed to 0.01% DMSO (control) or different doses of **(A)** concanamycin A (CCA) and **(B)** chloroquine (ChQ) for 24 hours, and cell viability was assessed using MTT assays. Data are expressed as the live cell fraction at each drug concentration relative to control. Error bars represent standard error of the mean (n=3). Red arrows indicate the 10 nM CCA and 50 µM ChQ doses used in these studies.

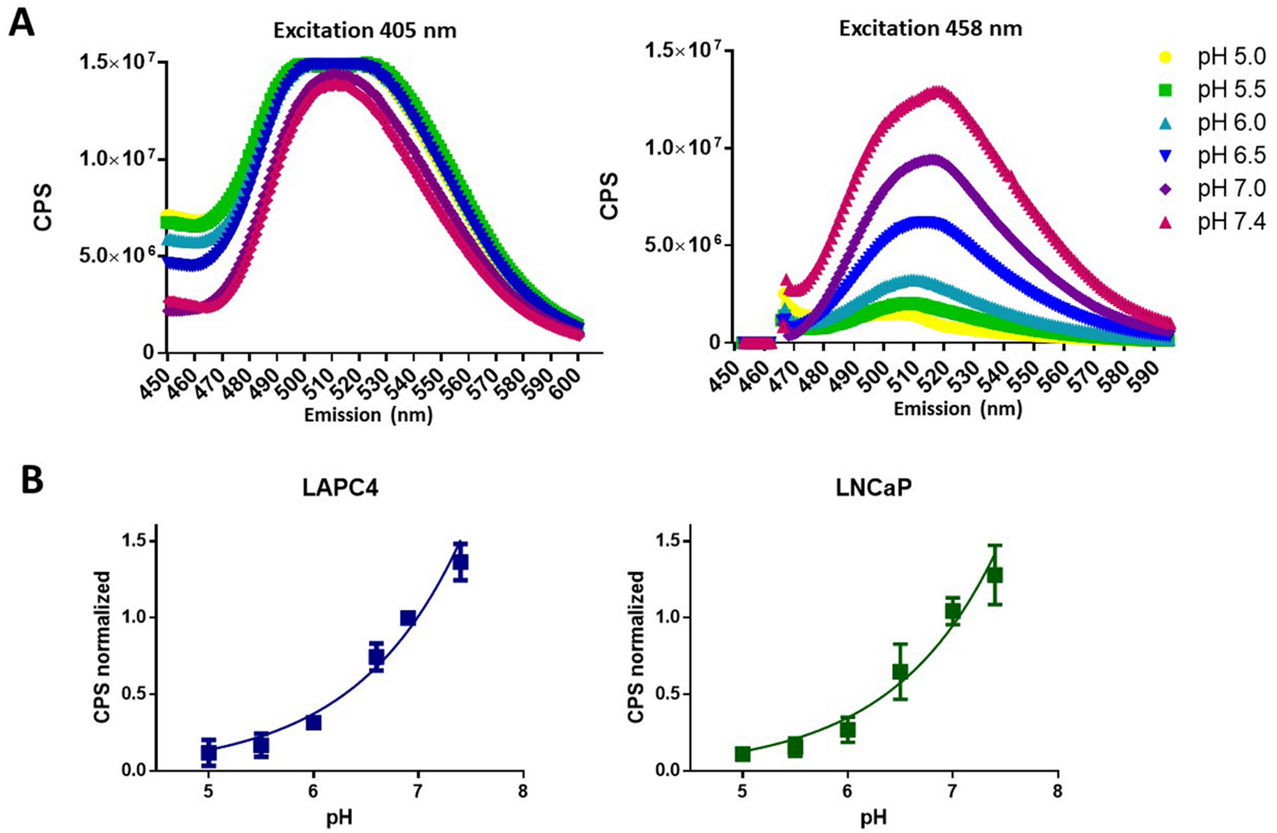

Supplementary Figure 2: HPTS pH-dependent spectra and standard curves. (A) Cells were incubated with 1 mM HPTS for ~16 hours, then equilibrated in different pH buffers (i.e., pH 5 to pH 7.4). Emission spectra (400-600 nm) were obtained at an excitation of 405 nm (left) and 458 nm (right). The representative spectra shown are from LNCaP cells. **(B)** LAPC4 and LNCaP cells were incubated with HPTS and then equilibrated in different pH buffers as described above. HPTS fluorescence (CPS) was measured at both 405nm excitation/515nm emission and 458nm excitation/515nm emission, and the 458/405 ratio (CPS normalized) was graphed as a function of pH. Both curves fit a non-linear regression (exponential growth equation) with a $R^2 > 0.95$.
